# Laparoscopic-assisted sclerotherapy in pediatric retroperitoneal lymphatic malformations

**DOI:** 10.3389/fped.2024.1418616

**Published:** 2024-07-08

**Authors:** Hao Shi, Zhibao Lv, Weijue Xu, Jiangbin Liu, Qingfeng Sheng, Xiang Ren, Zhou Chen

**Affiliations:** ^1^ Department of General Surgery, Shanghai Children's Hospital, School of Medicine, Shanghai Jiao Tong University, Shanghai, China; ^2^ Department of Radiology, Shanghai Children's Hospital, School of Medicine, Shanghai Jiao Tong University, Shanghai, China

**Keywords:** laparoscopy, lymphatic malformations, sclerotherapy, children, retroperitoneal

## Abstract

**Background:**

Retroperitoneal lymphatic malformations (LMs) are rare. Currently, the treatment of retroperitoneal LMs remains challenging. This study aimed to examine the safety and efficacy of laparoscopic-assisted sclerotherapy for retroperitoneal LMs in pediatric patients.

**Methods:**

We retrospectively reviewed patients treated with laparoscopic-assisted sclerotherapy for retroperitoneal LMs in a single tertiary medical center between July 2020 and February 2023. Doxycycline was prepared into a solution with a concentration of 10 mg/ml for use in sclerotherapy. Demographic data, clinical features, details of management, and outcomes were collected and analyzed.

**Results:**

A total of six patients, comprising three males and three females, were identified. The LMs were categorized into four macrocystic and two mixed-cystic types. The mean age and weight were 52.2 months (range, 11–108 months) and 20 kg (range, 12.5–27.5 kg), respectively. Three patients presented with abdominal pain or distension, while the other three patients were asymptomatic. All six patients underwent a total of eight sclerotherapy sessions. Two patients experienced intra-cystic hemorrhage and required a second sclerotherapy session. Only one patient presented with vomiting after sclerotherapy, which resolved spontaneously. Five patients met the complete response criteria, and one patient met the effective criteria. The mean reduction in lesion size was 92.3% (range, 69.9%–99.6%). No further complications or recurrence were recorded during follow-up.

**Conclusion:**

Laparoscopic-assisted sclerotherapy is a safe and effective approach for treating retroperitoneal LMs. This technique is applicable for both macrocystic and mixed-cystic retroperitoneal LMs.

## 1 Introduction

Lymphatic malformations (LMs) are common low-flow vascular malformations in children. The International Society for the Study of Vascular Anomalies (ISSVA) categorizes LMs into macrocystic, microcystic, and mixed-cystic types (1). Generally, LMs can occur anywhere lymphatic vessels are present. Abdominal lesions, including mesenteric, retroperitoneal, and omental LMs, account for only 3%–9.2% of all pediatric LMs (2). Among them, retroperitoneal LMs are particularly rare, accounting for <1% (3).

A well-established and mature treatment protocol has been formed for LMs in common locations such as the axilla, head, and neck. Surgery, oral medications, and sclerotherapy are the three main treatment methods, each with its limitations and advantages. Therefore, treatment options should be individualized for pediatric patients with LMs (4). Currently, the treatment of retroperitoneal LMs remains challenging due to their rarity. There is no conclusive evidence to determine the most optimal approach. The application of sclerotherapy in retroperitoneal LMs has been limited to small cohort studies and case reports. Herein, we present our experiences with laparoscopic-assisted sclerotherapy used for retroperitoneal LMs at our institution.

## 2 Methods

### 2.1 Study population

A retrospective review was conducted on children diagnosed with retroperitoneal LMs who were admitted for laparoscopic-assisted sclerotherapy in the General Surgery Department of Shanghai Children's Hospital between July 2020 and February 2023. All patients underwent ultrasound (US) and magnetic resonance imaging (MRI) before sclerotherapy. The lesions were classified as macrocystic (≥1 cm), microcystic (<1 cm), or mixed-cystic LMs, based on the cyst diameter measurements from images. Demographic data, clinical features, management details, and outcomes were collected and analyzed from medical records and online follow-up applications. The institutional review board of Shanghai Children's Hospital approved this study (2021R015).

### 2.2 Preparation

Doxycycline was used as a sclerosant for both primary and subsequent sclerotherapy sessions. The patient's weight was accurately measured upon admission. The doxycycline solution was prepared by dissolving 100 mg of doxycycline powder (Hainan General & Kangli Pharmaceutical Co., Ltd.) in 5 ml of saline and 5 ml of iohexol (Omnipaque, GE HealthCare Co., Ltd., Shanghai), resulting in a final concentration of 10 mg/ml. The maximum dose of doxycycline for a single procedure was limited to 20 mg/kg of body weight. The maximum length, width, and thickness of the lesions were measured on MRI images, and the intra-cystic fluid volume of the lesions was calculated using the ellipsoidal volume formula (*V* = 4/3πabc, *a* = 1/2 length, *b* = 1/2 width, *c* = 1/2 thickness). The volume of the infusion was set at half the volume of the aspirated fluid, referred to as the ideal dose. By comparing the maximum dose of doxycycline for a single procedure with the ideal dose, we then determined the dose each patient would receive for sclerotherapy.

### 2.3 Technique

**Figure 1:**
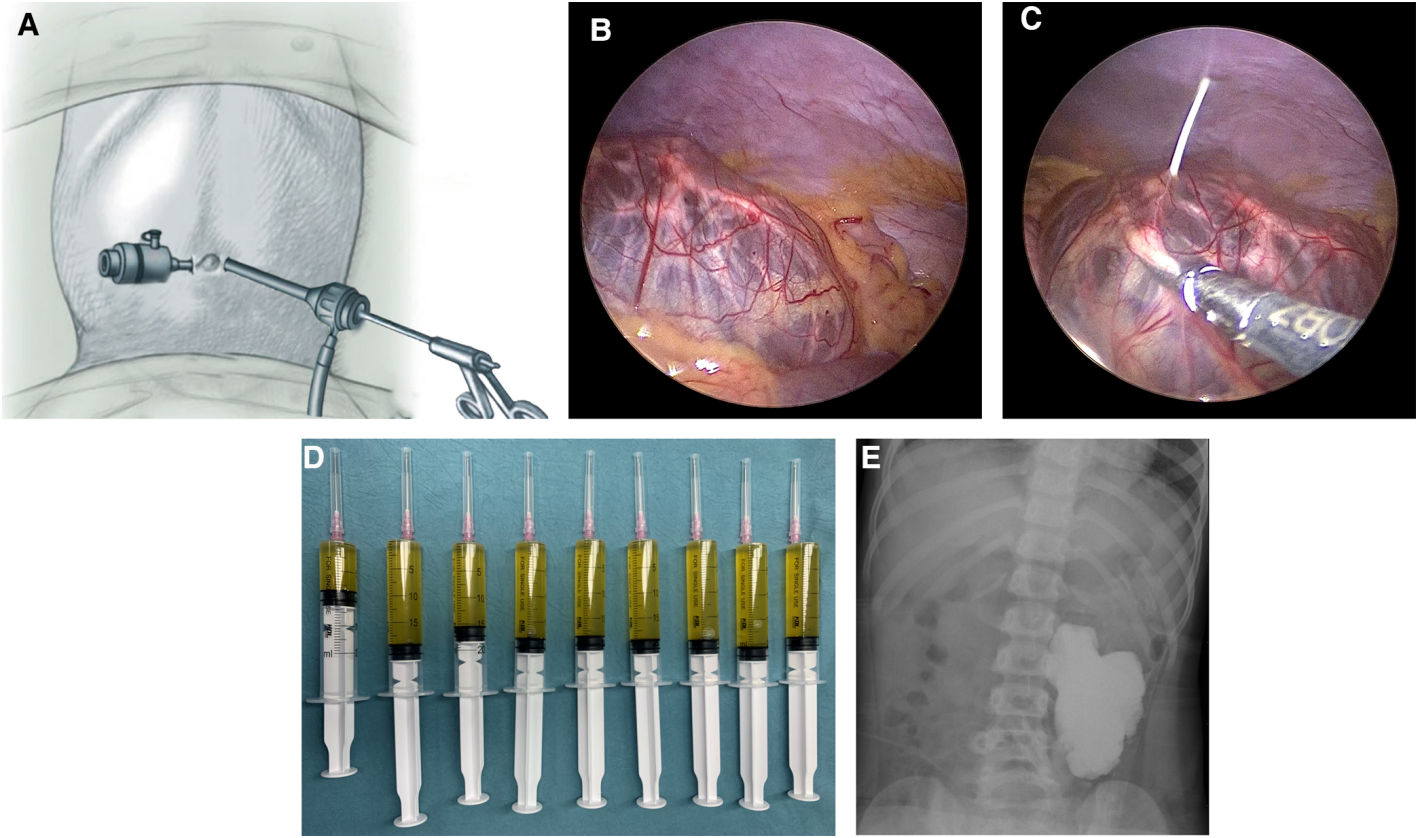
Steps of laparoscopic-assisted sclerotherapy. (**A**) Trocar arrangement. (**B**) Exploration of the location of the lesion. (**C**) Laparoscopic instruments assisted with the aspiration and infusion. (**D**) Aspirated fluid. (**F**) Bedside fluoroscopy.

All sclerotherapy procedures were conducted under general anesthesia. Initially, two paraumbilical incisions were made to insert two 3 mm trocars (Figure 1). Artificial pneumoperitoneum was maintained at a pressure of 8–12 mmHg, depending on the patient's age and weight. Laparoscopic instruments were used to explore the abdominal cavity and determine the precise location of the lesion (Figure 1). For lesions originating from the mesentery and omentum, if resection required sacrificing ≤10 cm of the intestine, surgical intervention was performed. Other lesions were treated with oral medications. The choice of surgical method, whether laparoscopic or open, depended on the surgeon's expertise. Patients who met the following criteria underwent laparoscopic-assisted sclerotherapy: (1) retroperitoneal macrocystic LMs and (2) retroperitoneal mixed-cystic LMs with macrocyst dominance (>50%). After confirming that lesions originated from the retroperitoneum, the flow and pressure of CO2 insufflation were reduced to minimize the distance between the lesions and the abdominal wall. A core needle was inserted through the abdominal wall into the cysts, and laparoscopic instruments were used to grasp the cystic wall and stabilize the needle (Figure 1). The needle direction was adjusted to ensure complete drainage. After aspiration, doxycycline was infused at the designated dose. A Hem-o-lok clip was used to clamp the puncture port on the cystic wall immediately after infusion. Bedside fluoroscopy was performed to assess the doxycycline coverage and observe potential drug leakage (Figure 1). The wounds were closed with tissue adhesive glue. No antibiotics were used during the perioperative period. The patients were hospitalized for observation and discharged after bowel function recovery.

### 2.4 Follow-up

The initial follow-up was conducted 6 weeks after the primary sclerotherapy. Subsequent follow-up time points were scheduled at 3 months, 6 months, and 1 year. Radiographic examinations were performed, including US at 6 weeks and 6 months and MRI at 3 months and 1 year. Efficacy was assessed by comparing the volume reduction of the lesions with the pre- and posttreatment volumes on MRI. The MRI images were analyzed by the same radiologist. Due to changes inside the lesions pre- and posttreatment, volume reduction was evaluated from different sequences at the same level.

Efficacy was categorized into three criteria, namely, complete response (≥90% reduction in volume), effective (50%–89% reduction in volume), and no response (<50% reduction in volume). When efficacy did not meet the effective standard or if the lesions enlarged during the initial follow-up, the lesions were observed for 1 month. Secondary sclerotherapy was performed if the lesions maintained their current status. Surgical or oral medication strategies were considered individually if the lesions showed no response after two sclerotherapy sessions.

## 3 Results

### 3.1 Demographics

From July 2020 to February 2023, a total of six patients with retroperitoneal LMs (three males and three females) underwent laparoscopic-assisted sclerotherapy in our center. The mean age of the patients at hospitalization was 52.2 months (range, 11–108 months), and the mean weight was 20 kg (range, 12.5–27.5 kg). Two patients presented with abdominal pain as the primary symptom, and one patient presented with abdominal distension. The lesions in three asymptomatic patients were incidentally detected by postnatal or antenatal US. Among the patients, four had macrocystic LMs, and two had mixed-cystic LMs. Patients’ characteristics are summarized in Table 1.

**Table 1:** Patients’ characteristics.

Patient	Sex	Age (months)	Weight (kg)	Symptom	Classification
1	F	60	21	Abdominal pain	Macrocystic
2	M	26	13	Abdominal distension	Macrocystic
3	F	60	26	Found incidentally by US	Macrocystic
4	M	48	20	Found incidentally by US	Mixed-cystic
5	F	108	27.5	Abdominal pain	Macrocystic
6	M	11	12.5	Found by antenatal US	Mixed-cystic

F, female; M, male.

### 3.2 Clinical characteristics

On pretreatment MRI imaging, the mean maximal diameter of the lesions was 11.7 cm (range, 6.0–19.7 cm). After MRI and laparoscopy were performed, the locations were determined, and all lesions were found to be unresectable. Although each patient underwent an MRI examination and received conclusions from one radiologist, laparoscopic localization ultimately revealed that the results differed from the preoperative imaging in four (66.7%) patients. Six patients underwent a total of eight sclerotherapy sessions, with two patients undergoing the procedure twice. The mean total dose of doxycycline infused in six patients was 454.2 mg (range, 60–840 mg). Three patients had to receive sclerotherapy based on the maximum dose of doxycycline allowed for a single procedure due to their weight limitations. Table 2 summarizes the detailed doses of doxycycline infused in all sessions.

**Table 2:** Detailed treatment information of patients.

Patient	Preoperative maximal diameter (cm)	Postoperative maximal diameter (cm)	Reduction of volume	Total dose (mg)	Maximum dose of single procedure (≤20 mg/kg)	Number of procedure	Efficacy	Complication
1	14.7	1.7	99.6%	840	420	2	Complete response	Intra-cystic hemorrhage; vomiting
2	11.8	6.1	96.5%	225	260	1	Complete response	None
3	9.0	2.5	99.1%	800	520	2	Complete response	Intra-cystic hemorrhage
4	6.0	3.4	69.9%	60	400	1	Effective	None
5	8.7	3.2	93.6%	550	550	1	Complete response	None
6	19.7	8.4	95.2%	250	250	1	Complete response	None

### 3.3 Outcomes

**Figure 2:**
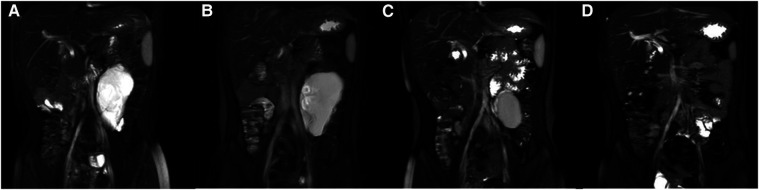
Pre- and posttreatment changes of lesions on MRI (Case 1). (**A**) Pretreatment MRI. (**B**) Posttreatment MRI 1 month after the first sclerotherapy. (**C**) Posttreatment MRI 6 months after the second sclerotherapy. (**D**) Posttreatment MRI 1 year after diagnosis.

Comparing the preoperative and posttreatment volumes of lesions on MRI, the mean posttreatment maximal diameter of the lesions was 4.2 cm (range, 1.7–8.4 cm), and the mean reduction in size was 92.3% (range, 69.9%–99.6%). Eventually, five patients met the complete response criteria, and only one patient met the effective criteria, whose lesions were of the mixed-cystic type, and received only 60 mg doxycycline. A typical case (Case 1) is presented in Figure 2. One patient presented with vomiting 1 day after sclerotherapy, which resolved spontaneously later. No fever was recorded, and CBC conducted 3 days after surgery in these patients indicated normal levels of both white blood cells and C-reactive protein (CRP). During follow-ups, no response was observed in two patients due to intra-cystic hemorrhage after sclerotherapy. Although sclerotherapy induced intra-cystic hemorrhage, hemoglobin levels did not exhibit significant changes. These two patients were readmitted later for a second sclerotherapy. No further complications or recurrence were recorded.

## 4 Discussion

Lymphatic malformations (LMs) are common diseases in the pediatric population. Macrocystic, microcystic, and mixed-type are the three categories of LMs, classified based on the diameter of cysts, with cutoffs of 1 cm or 2 cm (5). The head and neck are the most commonly affected areas, accounting for approximately 50% of all LMs (6). In contrast, retroperitoneal LMs are extremely rare, accounting for <1% of all LMs (3). The clinical manifestation of LMs is closely related to their size and location. Patients with retroperitoneal LMs may present with abdominal pain, distension, vomiting, and hematuria due to the compression and obstruction of surrounding organs or tissues (7). However, these symptoms are non-specific and can be observed in other pediatric acute abdominal diseases. A minority of patients are asymptomatic due to the large carrying capacity of the abdominal cavity, leading to incidental diagnosis during examinations (3).

In recent years, the management of retroperitoneal LMs has gradually gained the attention of pediatric surgeons (2, 7–9). However, current clinical management relies on personal experience and lacks national or worldwide standardized guidelines due to the low incidence. Challenges in management remain. The main methods include surgery, sclerotherapy, oral medication therapy, and observative treatment (10). Management requires the coordinated efforts of specialists in both medical and surgical fields and should be tailored to the size, location, symptoms, and category of the lesions (5).

For those who are asymptomatic, conducting observative treatment is based on the possibility that some lesions may regress spontaneously and the desires of the patient's family (11). This treatment, previously reported for head and neck LMs, should be individualized after stratifying by de Serres stage and confirming that the lesions are non-function threatening (12). However, observation treatment is not suggested in most reports due to the low regression rate and limited reduction size (12). In our study, we intervened in three asymptomatic patients after diagnosis. We point out that as the children grow older, the size of lesions may increase and patients may encounter risks of complications such as infection, rupture, and compression on surrounding organs and tissues (13, 14).

Surgery was the only treatment option in the past. However, for giant retroperitoneal LMs, complete resection by an open or laparoscopic approach can be challenging. The deep location, unclear boundaries of retroperitoneal LMs, and organ invasion increase the difficulty of complete resection, and incomplete resection results in possible recurrence (15). There are intraoperative conditions and postoperative complications, such as damage to vessels and organs, lymphatic leakage, slow recovery of intestinal function, adhesive intestinal obstruction, and aesthetically affected scars. Redo surgery would be even more difficult. Postoperative complications increase psychological stress and financial burden on caregivers. Surgery is no longer considered the first-line treatment now but remains irreplaceable in certain cases, including lesions resistant to sclerotherapy and oral medication therapy, and life-threatening conditions such as spontaneous rupture, obstruction, severe intra-cystic infection, and abdominal compartment syndrome (16–18).

Sclerotherapy is recognized as the first-line treatment for head and neck LMs (19). There are multiple options for sclerosing agents, with doxycycline being widely used due to its low cost, widespread availability, high efficacy, and safety in macrocystic LMs (20–22). Sclerotherapy is more effective in treating macrocystic LMs compared with microcystic LMs because large cysts are much easier to aspirate and infuse (11). Retroperitoneal LMs commonly present with large size and are predominantly macrocystic (3). Therefore, we only performed laparoscopic-assisted sclerotherapy on patients diagnosed with retroperitoneal macrocystic LMs or mixed-cystic LMs with macrocyst dominance (>50%). Patients diagnosed with retroperitoneal microcystic LMs were managed with oral medication therapy in our study. Sclerotherapy can be performed under US or fluoroscopy. These methods may not be suitable for deep lesions or for those in anatomic locations that might be difficult to repeat treatments, for example, retroperitoneum (21). In addition, although imaging plays an important role in diagnosis, it is challenging to differentiate other abdominal LMs from retroperitoneal LMs. Therefore, in this study, we used laparoscopy to confirm the lesion's location and assist with sclerotherapy. There is no need to perform sclerotherapy on omental LMs and mesenteric LMs that invade short bowel segments because complete resection under laparoscopy has a good clinical response (2, 8). Laparoscopic exploration allows surgeons to change treatment options in such circumstances. During sclerotherapy, laparoscopy provides a clear view to monitor and allows laparoscopic instruments to stabilize both the cystic wall and the needle tip. For some LMs with several cysts, it is difficult to confirm which cysts have been completely aspirated and infused using indirect monitoring methods. Additionally, needle tips may adhere to the cystic wall during aspiration, leading to the misconception that complete aspiration has been finished. In contrast, laparoscopy can determine whether the cysts have undergone sclerotherapy by monitoring the volume changes and internal fluid flow of a single cyst. Previous studies have shown that thorough aspiration of intra-cystic fluid is a crucial factor influencing the effectiveness of sclerotherapy (23).

Oral medication therapy has become a research hotspot in the past decade and is considered the first-line therapy for microcystic LMs (24). Research on the pathogenesis of LMs has promoted the development of oral medications, such as sirolimus and alpelisib, targeting the PI3K/Akt/mTOR signaling pathway (25, 26). However, oral medication therapy requires a long period of time and relies heavily on compliance and supervision. To maintain the therapeutic concentration, plasma levels should be periodically monitored, and doses should be adjusted as the patient grows (24, 27). Currently, there is no established standard for the optimal plasma concentration and the appropriate time point for discontinuation. More research is needed to focus on long-term safety and efficacy and to standardize the treatment.

In this study, five patients met the complete response criteria. Among them, three patients were not treated with half the volume of the aspirated fluid because the dose was limited by weight. Based on our past experience, they may need to undergo multiple sclerotherapy sessions, but except for Case 1, who experienced posttreatment intra-cystic hemorrhage, all other patients had a single sclerotherapy and presented a good clinical response. This may be related to the complete aspiration of intra-cystic fluid under laparoscopy. One patient met the effective criteria because the lesions were of mixed type, the microcystic part showed a poor response to sclerotherapy. Additionally, we also focused on whether radical resection is necessary following a good response to sclerotherapy. We conducted follow-ups over 6 months to 2 years, during which radiological examinations revealed no recurrence. The patients were asymptomatic, leading us to decide on monitoring and observation instead of radical resection.

Posttreatment complications of sclerotherapy in retroperitoneal and other abdominal LMs are well described with an incidence ranging from 10% to 20% (28). Intra-cystic complications include hemorrhage and infection, while extra-cystic complications include lymphaticocutaneous fistula, extravasation of doxycycline, and bowel perforation and obstruction (2, 7, 29, 30). However, in current literature, sclerotherapy is typically performed under US or fluoroscopy. Assisted by laparoscopy, a direct monitoring method, it can effectively prevent needle dislodgement and drug leakage. Laparoscopy also plays a crucial role in mitigating the risk of inadvertent injury to surrounding tissues when the needle moves (29). Some extra-cystic complications, such as extravasation, lymphaticocutaneous fistula, and bowel perforation, may be avoided with the use of laparoscopy. In our series, one patient experienced vomiting after sclerotherapy, which resolved spontaneously, and two patients had intra-cystic hemorrhage detected at the first follow-up. We observed these two patients for an additional 1 month and reperformed laparoscopic-assisted sclerotherapy. These two patients eventually met the complete response standard.

The limitations of our study include its retrospective design and small sample size. Further multicenter studies are needed to examine the long-term efficacy and safety of this treatment for retroperitoneal LMs. In addition, we measured the diameters of lesions on cross-sectional MRI and used the ellipsoidal volume formula to evaluate pre- and posttreatment changes. There may be some deviation because the shapes of LMs are typically irregular, which may influence outcome evaluation. More precise evaluative methods should be used in the future.

## 5 Conclusion

Laparoscopic-assisted sclerotherapy for retroperitoneal LMs is both effective and safe. This procedure can be used as an initial intervention or as an alternative option when complete resection is challenging. Laparoscopy aids in precisely locating the lesion and stabilizing the aspiration and infusion process, thereby enhancing the accuracy of sclerotherapy and reducing posttreatment complications.

## Data Availability

The raw data supporting the conclusions of this article will be made available by the authors, without undue reservation.
